# Pathway Analysis of Differentially Expressed Genes in Patients with Acute Aortic Dissection

**DOI:** 10.4137/bmi.s2530

**Published:** 2009-05-06

**Authors:** Salah A. Mohamed, Hans H. Sievers, Thorsten Hanke, Doreen Richardt, Claudia Schmidtke, Efstratios I. Charitos, Gazanfer Belge, Joern Bullerdiek

**Affiliations:** 1 Department of Cardiac Surgery, University Clinic of Schleswig-Holstein, Campus Luebeck, Luebeck, Germany; 2 Center for Human Genetics, University of Bremen, Bremen, Germany

**Keywords:** acute aortic dissection, marfan syndrome, microarrays, pathway analysis

## Abstract

**Background:**

Acute aortic dissection (AAD) is a life-threatening condition with high mortality and a relatively unclarified pathophysiological mechanism. Although differentially expressed genes in AAD have been recognized, interactions between these genes remain poorly defined. This study was conducted to gain a better understanding of the molecular mechanisms underlying AAD and to support the future development of a clinical test for monitoring patients at high risk.

**Materials and Methods:**

Aortic tissue was collected from 19 patients with AAD (mean age 61.7 ± 13.1 years), and from eight other patients (mean age 32.9 ± 12.2 years) who carried the mutated gene for Marfan syndrome (MS). Six patients (mean age 56.7 ± 12.3 years) served as the control group. The PIQOR^TM^ Immunology microarray with 1076 probes in quadruplicates was utilized; the differentially expressed genes were analysed in a MedScan search using Pathway Assist software. Quantitative reverse transcription-polymerase chain reaction (qRT-PCR) and protein analysis were performed.

**Results:**

Interactions of MS fibrillin-1 (FBN1) in the MedScan pathway analysis showed four genes, *fibulin-1* (FBLN1), *fibulin-2* (FBLN2), *decorin* (DCN) and *microfibrillar associated protein 5* (MFAP5), which were differentially expressed in all tissue from AAD. The validation of these genes by qRT-PCR revealed a minimum of three-fold downregulation of FBLN1 (0.5 ± 0.4 vs. 6.1 ± 2.3 fold, *p* = 0.003) and of DCN (2.5 ± 1.0 vs. 8.5 ± 4.7 fold, *p* = 0.04) in AAD compared to MS and control samples.

**Conclusions:**

Downregulation of fibrillin-1 (FBN1) may weaken extracellular components in the aorta and/or interfer with the transmission of cellular signals and eventually cause AAD. Additional research on these four identified genes can be a starting point to develop a diagnostic tool.

## Introduction

Acute aortic dissection (AAD) is a life-threatening clinical entity requiring urgent surgical intervention. The peak incidence of aortic dissection is reported to occur in the sixth and seventh decades of life with 2000 new cases per year in North America and 3000 in Europe.^1^,^2^

The initiating event is an intimal tear due to congenital defects of the aortic wall, such as in Marfan syndrome (MS), Ehlers–Danlos syndrome (a heterogeneous group connective tissue disorders), idiopathic Erdheim Gsell medial necrosis, bicuspid aortic valve (the most common congenital heart disease), or even acquired intima damage caused by hypertension, thoracic trauma or other unknown mechanism.^3^–^5^ Subintima or media necrosis results in intimal tear, which eventually allows blood to enter into the aortic wall, thus leading to separation of the aortic wall layers and hence to a rapid propagation of the aortic dissection. The separation of the media and the adventitia layers of the aortic wall may lead to malperfusion of numerous organs with devastating complications.^2^,^6^

An important clinical fact is that aortic dissections occur in both aneurysmatic expanded calibre as well as in normal calibre of the aorta. The most feared complication of aortic dissection is its outward rupture, which is associated with high mortality.^7^ The surgical treatment depends on the location of the initial tear and on the longitudinal propagation of the dissection. To describe dissections various classifications have been proposed, with the DeBakey and the Stanford classification more often used.^8^,^9^

When the ascending aorta is affected (Stanford type A), the mortality of untreated patients is about 36%–72% within the first 48 hours and 62%–91% within the first week.^10^–^13^ Chest pain and other symptoms of ADD are often confused with those of cardiac infarction, thus complicating the diagnosis and delaying the treatment of AAD. Therefore, a better understanding of the molecular mechanisms underlying AAD can be the first step to supporting the future development of a rapid test for monitoring patients at high risk.

Questions about the underlying mechanisms in the aorta that make it more prone to be dissected and why many patients without gene defects on *fibrillin-1* (FBN1) can be at high risk of developing AAD remain unanswered. The aim of this study was to investigate whether aortic tissue from patients with AAD presents significant similarities in their gene expression patterns and to compare these findings with those from known MS. Using pathway analysis, the analysis of relationships between genes/proteins and the processes or diseases in which these genes/proteins are involved, was used to show which of the differentially expressed genes in AAD can interact with MS FBN1.

## Materials and Methods

The study protocol was approved by the institutional ethics committee and written informed consent from all patients was obtained. In cases of ascending aorta replacement in patients with type A aortic dissection (AAD) or with Marfan syndrome (MS) without acute dissection, diseased aortic tissue was collected during surgery and immediately frozen in liquid nitrogen then used later for gene expression and protein analysis. To minimize the impact of age differences between patients with AAD and MS on the confidence interval, additional tissue was taken during valve replacement surgery (Ross procedure, normal aorta and aortic valve) from six patients and used as control. Patients’ demographics are shown in Table 1.

## Patients and Work Program

Samples were collected from whole aorta of 19 patients with AAD (dissection type A, mean age 61.7 ± 13.1 years), eight patients with phenotypic features of MS who demonstrated *fibrillin-1* gene mutation (mean age 32.9 ± 12.2 years). In addition, aortic wall tissue samples taken during Ross procedure in cases of normal aorta (aortic diameter ≤ 37 mm) and without malformation of the aortic valve from six patients (mean age 56.7 ± 12.3) were used as control. 5 ml EDTA venous blood for screening of mutations on *fibrillin-1* gene were collected. Microarrays were hybridized to detect differentially expressed genes between patients with MS (labeled with Cy3) and patients with AAD (labeled with Cy5). The differentially generated genes of interest from these microarray results were validated for all patients of this study in the quantitative reverse transcription-polymerase chain reaction (qRT-PCR). Protein anlaysis was also performed for the most significantly expressed genes.

### RNA extraction

Total RNA was isolated using standard RNA purification protocols (Trizol; Sigma Aldrich, Germany). The integrity of total RNAs was checked via the *Bioanalyzer 2100* system (Agilent Technologies). The peak areas of 28 S and 18 S RNA of every sample was determined and the ratio of 28 S/18 S calculated.

### RNA amplification and labelling

For hybridizations on PIQOR^TM^ microarrays, linear amplification of RNA was performed using a modified methodology described elsewhere.^14^ Amplified RNA (aRNA) samples were quantified by spectrophotometry and the quality of the samples was proofed by gel electrophoresis (Bioanalyser 2001; Agilent). One microgram of aRNA from diseased tissue of AAD versus tissue of MS was labeled by reverse transcription with Cy5 and Cy3 fluorescence, respectively. According to this study design, samples from normal aortic tissue were labeled by reverse transcription with Cy5. Labeled samples were then hybridized on PIQOR^TM^ Immunology microarrays.

### Bioinformatics service: Re-calculation of new expression ratios based on a new reference

In all microarray experiments the samples obtained from MS had been used as reference. To be able to compare all expression values with another control of normal aortic tissue, we labeled by reverse transcription every new reference with Cy5 and hybridized against the common MS. We performed in first microarray experiments compared AAD against MS the expression ratios of these experiments were set against the expression ratio of second microarray experiments compared normal aortic tissue against MS.

### Microarray production

Microarray production was performed as described previously.^15^ Briefly, prespecified 200–400-bp fragments of selected cDNAs were generated by RT-PCR (Superscript^TM^II; Invitrogen, Groningen, The Netherlands), cloned into pGEM^®^-T Vector (Promega, Mannheim, Germany) and sequence-verified.^16^ Amplified inserts (Taq PCR Master Mix; Qiagen) were purified (Qiaquick 96 PCR BioRobot Kit; Qiagen), checked on an agarose gel, and spotted four times each (0.2 ng) on treated glass slides.

### Array hybridization and data analysis

Hybridization, scanning and data analysis were performed as described in detail elsewhere.^12^ Briefly, image capture and signal quantification of hybridized PIQOR^TM^ microarrays were performed with the ScanArraylite (Packard Bioscience, Billerica, MA, U.S.A) and ImaGene software Version 4.1 (BioDiscovery, Los Angeles, CA, U.S.A). Local background was subtracted from the signal to obtain the net signal intensity and the ratio of Cy5/Cy3. Subsequently, the mean of the ratios of four corresponding spots representing the same cDNA was computed. For normalization, only the spots for which the fluorescent intensity in one of the two channels was at least two times the mean background intensity of all unflagged spots was used. Only the genes displaying a net signal intensity two-fold higher than the mean background were used for further analysis.

### Identification of differentially expressed genes

As a rule of thumb, changes in expression in the PIQOR microarray experiments of more than twofold are considered to be reliable and significant. Thus, all genes in our lists were with log 2 ratio values equal and above 1.0 and equal and lower than −1.0. The various lists were compared and genes which appeared on all the compared lists were compiled in new sublists. This provided a condensed list of genes that contain only those genes differentially expressed in all the analysed samples.

### Pathway analysis of the common differentially expressed genes

Pathway analysis means the analysis of relationships between genes or proteins and the processes or diseases in which these genes or proteins are involved. In order to get an idea about the biological meaning of the differential expression of the common up- and downregulated genes, differentially expressed genes were subjected to biological pathway analysis.

### Interactions with fibrillin-1 using a MedScan search and pathway assist software

A MedScan search was performed and followed by using Pathway Assist software (www.ariadnegenomics.com) to look for interactors of *fibrillin 1* gene. This software automatically screens databases of scientific abstracts via the Internet and collects recognized biological terms, matching and filtering them in reference to the user’s input. The software defines descriptions of sorted gene/protein functions and interactions, regulatory events, biochemical cellular pathways and links between them. It provides a built-in database, containing thousands of events of regulation, interaction and modification between proteins, cell processes and small molecules, compiled by the application of text- and data-mining software to PubMed. This tool assists users to define the common regulators and targets of differentially expressed genes.

### Validation of the common differentially expressed genes using qRT-PCR and protein analysis

Validation using qRT-PCR of 30 genes was undertaken for all of the 33 patients in this study. Total RNA was prepared using RNeasy^TM^ Total RNA Kit (Qiagen, Hilden, Germany). Primers are listed in Table 2; run conditions and measurement have been described previously.^17^ For protein analysis, 100 mg of tissue were homogenized in 500 μl lysis buffer. SDS-PAGE (10%) and transferred to a nitrocellulose membrane for Western blot analysis according to the manufacturer’s instructions (Amersham Pharmacia). The polypeptide bands were semi-quantified using computerized blot scanning; the optical density of every sample was estimated after the normalization to anti beta Actin (Acris Antibodies GmbH, Hidenhausen, Germany) and compared to control sample using ImageJ (http://rsb.info.nih.gov/ij/).

### Statistics

All data are presented as mean ± SD. Comparison was made between type A (acute dissection) versus Marfan syndrome and between Marfan syndrome versus control using standard Student’s *t*-test. Differences among groups were assumed significant at p values <0.05.

## Results

### Inter-experiment correlation analysis

**Figure 1:**
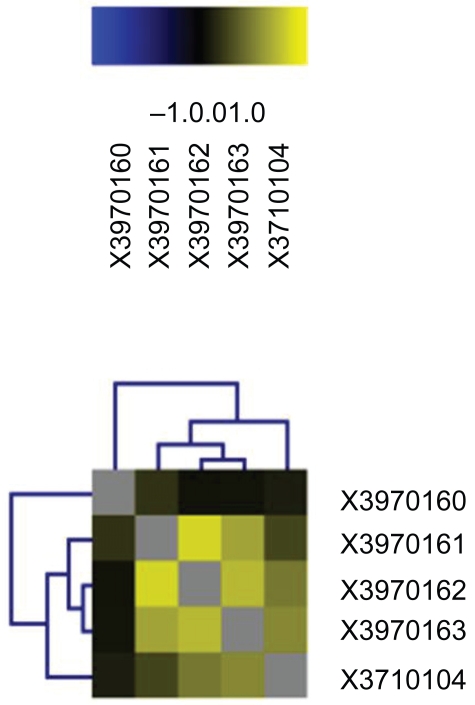
Global inter-experiment correlations analysis. Representatively, detection of gene expression: lanes with the initials X3970160, X3970161, X3970162, X3970163 demonstrated the detection of gene expression on microarray, RNA obtained from four samples of AAD tissue hybridized against RNA obtained from MS tissue. In lane X3710104 pooled RNA of eight patients with AAD is shown.

The global expression profiles of microarrays were compared in a correlation analysis; the obtained inter-experiment correlation coefficients were listed. The various experiments were plotted against each other in scatter diagrams to visualize the correlation between the samples, followed by a graphical representation of the correlation values. Figure 1 demonstrated the results of five microarrays (AAD versus MS). Positive correlations are shown in shades of yellow (more intensive colour = better correlation), while anti-correlations are indicated by shades of blue.

### Identification of differentially expressed genes

Genes with more than a two-fold change in expression in the PIQOR microarray experiments were considered to be reliable and significant, and divided according to their functions in lists. The different lists were compared, and genes which appeared in all of the compared lists were compiled into new sublists. A condensed list of 16 genes (Table 3) was created containing only those genes which were differentially expressed in all the analysed samples.

In an additional approach, the mean values of the expression values of all the genes were calculated and those with an at least two-fold up- or downregulation were extracted to a new list. This method is less stringent and allows genes with missing expression values for one or more experiments to be considered for later pathway analysis. The list obtained with the latter method contained 88 genes, generated from the medians of the gene expression, which were clustered according to their biological annotations. A group of 32 genes with high significance, involved in extracellular matrix assembly and in extracellular space building was identified (shown in Table 4). The expression of genes with high significance was validated in qRT-PCR with primers listed in Table 2, and then protein analysis was performed.

### Pathway analysis of the common differentially expressed genes

The genes which were differentially expressed in all analysed samples (listed in Table 4) were subjected to biological pathway analysis, revealing another major group (clusters). A closer look at these clusters revealed proteins involved in extra-cellular matrix assembly or maintenance as well as cell adhesion and signalling.

A number of matrix metalloproteases (MMP-19, MMP-12, MMP-9) were differentially expressed in tissues obtained from patients with AAD. MMP-19 was the only matrix metalloprotease being upregulated, while MMP-12 and MMP-9 were downregulated. Furthermore, the expression levels of some collagens were reduced (COL11A1, COL1A1, COL3A1, COL1A2, COL15A1). Among the seven chemokines which were differentially expressed in the microarrays, six were downregulated in their expression. Only CCL2 was upregulated by a factor of 2 to 3 in four of the five microarrays. The other chemokines (CCL13, CCL14, CCL15, CXCL14, CCL21 and CCL19) were all downregulated in the analysed samples. In addition, IL6 was upregulated.

### Interactions with fibrillin-1 (FBN1)

**Figure 2:**
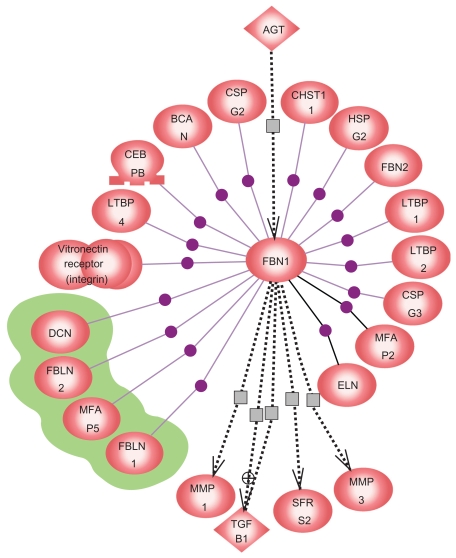
Interactors of FBN1 found by a MedScan search using Pathway Assist software. The genes coding for the proteins with the green halos were differentially expressed in the current microarray experiment.

Interactors of FBN1 revealed by a MedScan search using InteractionExplorer software Pathway Assist showed four genes (*fibulin-1* (FBLN1), *fibulin-2* (FBLN2), *decorin* (DCN) and *microfibrillar associated protein 5* (MFAP5)), which were differentially expressed in all AAD patients included in the analysis (Fig. 2).

We used the InteractionExplorer software again to check all the differentially expressed genes for further analysis to study interactions and molecular networks. Among the regulators of the differentially expressed genes certain growth factors were identified; PGF, EGF, TGFB1, HGF and platelet-derived growth factor.

### Validation of the common differentially expressed genes using qRT-PCR and protein analysis

**Figure 3:**
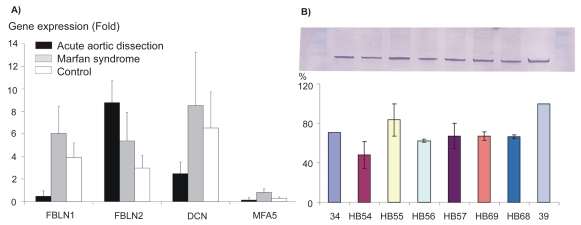
Validation of four differentially expressed genes using qRT-PCR is presented in **3A**; those four genes are directly interact in the pathway analysis with FBN1. They are involved in ECM development. Representatively, in **3B**) a Western blot demonstrated detection of *fibulin-1* protein in tissue obtained from Marfan syndrome (34), in six different patients with AAD (HB54,-55,-56,-57,-69 and HB68) and in control (39).

qRT-PCR, after three repeats, revealed a minimum three-fold downregulation of FBLN1 (0.5 ± 0.4 vs. 6.1 ± 2.3 fold, *p* = 0.003) and of DCN (2.5 ± 1.0 vs. 8.5 ± 4.7 fold, *p* = 0.04) in AAD compared to MS and control samples. Representatively, validation of the four interactions of FBN1 is shown in Figure 3A. The average of FBLN1 protein in all investigated AAD samples after the normalization to Actin and semi-quantified, showed 30% downregulation (*p* ≤ 0.02), Figure 3B demonstrates the detection of FBLN1 protein in eight representative samples.

## Discussion

Despite its high mortality rate, acute aortic dissection (AAD) presents a relatively unclarified entity of the arterial system. It was first described in the eighteenth century during autopsy by Dr. Nicholls, physician to King George II.^18^ Even today AAD remains a disease with high mortality rates, with about 2000 new cases in North America and 3000 new cases in Europe every year. It requires urgent clinical intervention; untreated cases have a mortality rate reaching about 36%–72% within the first 48 hours (classification after Stanford type A).^9^

In their study Weis-Müller et al described the molecular features underlying the pathogenesis of AAD as obscure.^10^ They compared tissue from dissected aorta with normal aortic tissue using microarrays and found some indications of AAD differentially expressed genes;^19^ however, the pathway of these differentially expressed genes is still to be defined. Also, the underlying mechanisms which make the aorta more prone to be dissected remain unclarified. In this study we investigated whether dissected aortic tissues present significant similarities in their gene expression pattern. We generated 88 differentially expressed genes in tissue obtained from patients with AAD compared with MS by using microarrays, and clustered them according to their biological annotation. We found that most of the genes identified as differentially expressed are assigned to ‘extracellular space’. Examination of these clusters revealed proteins involved in extra-cellular matrix assembly or maintenance, as well as cell adhesion and signalling.

A number of matrix metalloproteases (MMP-19, MMP-12, MMP-9) was differentially expressed in AAD patients. MMP-19 was upregulated and MMP-12 and MMP-9 were downregulated. The expression levels of some collagens were reduced (COL11A1, COL1A1, COL3A1, COL1A2, COL15A1). Our results suggest that the extracellular matrix was weakened and cell adhesion might be reduced in affected patients.

Seven chemokines were identified; six were downregulated in their expression. Only CCL2 was upregulated, by a factor of 2 to 3. CCL2, a potent leukocyte chemoattractant, is known to induce macrophage recruitment and activation involved in diseases such as psoriasis, rheumatoid arthritis and arteriosclerosis.^20^–^22^ In addition, IL6 was upregulated, indicating that the tissues of the affected patients presented an inflammatory response in AAD. Another interesting gene that we found (data published elsewhere) with high expressed in AAD (not included in the PIQOR microarray) is the high mobility group AT-hook 2 (*HMGA2*).

The investigated AAD patients presented neither clinical manifestation nor mutations of *Fibrillin-1* gene, however, expression levels of *Fibrillin 1 gene* and its interaction partners in AAD samples are of interest. A reduced expression of *Fibrillin-1* and its interaction partners could be an alternative molecular mechanism leading to AAD phenotype. It is known that fibrillin-1 protein can bind fibulin-1 and -2 as well as decorin. Downregulation of *fibulin-1* gene in AAD corresponds with its possible impact on this disease. Fibulin-1 is an essential component of vascular basement membrane, fibulin-1 knockout mice die soon after birth due to massive bleeding from small sized vessels.^23^ At the same time, there is evidence about a regulatory function of fibulin, as it inhibits the activity of the extracellular signal-regulated kinase, and thus the signal transduction.^24^ An altered *Fibulin-1* gene expression might be a decisive factor in the formation of aortic dissection in Patients without Marfan-Syndrome.

Therefore, a downregulation of fibulin-1 expression may contribute to the development of aortic dissection, either by weakening the aortic connective tissue or by altering the cellular signal transduction. Decorin is considered as another candidate gene for Marfan syndrome.^25^ It is interesting to see *fibulin-2* is upregulated in AAD. The upregulation expression of *fibulin-2* is in AAD probably due to multiple different reasons (inflammation, hemodynamic effects-high blood pressure, etc.). Low expression levels of decorin may lead to restriction in the ability to assemble extracellular matrix and to build up connective tissue in the aorta, with the consequence of presenting the AAD phenotype. We conclude that the downregulation of *fibulin-1* and/or of decorin in AAD patients could be an alternative molecular mechanism—opposed to a mutation in *fibrillin-1* as seen in patients suffering from Marfan syndrome—leading to the same or similar phenotype. Further studies of decorin, fibulin-1, and others like HMGA2 may prove their role and how they may develop the symptoms of AAD. Since a clinical test to detect AAD on a daily basis is still lacking,^26^ these results may increase our understanding of the molecular mechanisms underlying AAD, and thus may support the future development of a panel of markers as a clinical test for monitoring patients at high risk.

## Figures and Tables

**Table 1:** Patients’ demographics.

Definition	N	Age (Y)	BMI (Kg/m^2^)	LDH (U/L)
AAD	19	61.7 ± 13.1	27.0 ± 3.0	162.7 ± 60.6
MS	8	32.9 ± 12.2	23.0 ± 3.5	213 ± 155.8
C	6	56.7 ± 12.3	28.3 ± 3.3	210.0 ± 67.3

**Abbreviations:** AAD, acute aortic dissection; MS, marfan syndrome; C, further control without dilation of the aorta.

**Table 2:** Primer sequences.

Gene symbol	Forward primer	Reverse primer	Fragment size (bp)
FBLN1	TCA TCT CGC TGC CTA CCT TC	CCG TCC ATG TAA CGC TTG AT	163
FBLN2	TCA CGC ACT ACC AGC TCA AC	CTG CCT CCA GAG CTT CAT CT	239
DCN	ATG TGT CTA CGT GCG CTC TG	CTG AAA ATG GCA GGC AAA AT	241
MMP19	ACA GCC CTT CCA ACT GTG TC	TGC CCG TAA TCA TAA GCA CA	166
IL6	TAC CCC CAG GAG AAG ATT C	TTT TCT GCC AGT GCC TCT TT	175
CCL2	TCC CCA GAC ACC CTG TTT TA	CAA AAC ATC CCA GGG GTA GA	199
MFAP5	GCT TCA CCA GTT TAC GAC GTA TG	ACA GGG AGG AAG TCG GAA GT	161
MFAP2	TCC TGT GCC AGG AGC TG	GGG CTG CAG TCC ACT AAC TT	157
EGR1	CTG GTG GAG ACC AGT TAC CC	TGG GTT GGT CAT GCT CAC TA	156
ITGB4	GCC TTC ACT TTG AGC ACT CC	ACT TGT AGG GCA CGT TCT CG	238
THBD	ACA TCG ACG AGT GCG AAA AC	GGA GAT GCC TAT GAG CAA GC	245
ITGA5	AAG CCC TGA AGA TGC CCT AC	GGA GCG TTT GAA GAA TCC AA	203
TGFB1	CAA CAA TTC CTG GCG ATA CC	GAA CCC GTT GAT GTC CAC TT	193
CCL13	GCT GGC AGT GGG TTT GTA TT	TTG CAT TCA TCT TTC CAC AA	152
CXCL14	GGT CCA AAT GCA AGT GCT C	CAG TGC TCC TGA CCT CGG TA	151
CCL14	CAA CCC CAG TGA CAA GTG G	AAG CTC CAA GAG GGT GAC TG	166
CCL15	CAC ATC CCA ATC CTG AAT CC	CAA GGC TGA GAG TGC AAC AG	205
PRG4	TGC CAG AAT TGA ACC CTA CC	CTT CAG GCA TGA ACA CAT GG	177
TGFBR2	CTA ACC TGC TGC CTG TGT GA	TCG GTC TGC TTG AAG GAC TC	167
MMP9	GGG AAG ATG CTG CTG TTC A	TCA ACT CAC TCC GGG AAC TC	202
GEM	ATG CAG CCA CAG CAG CAG	GTC TGT GGA GTC AGA GGA CCA	150
SFRP2	GCC TCG ATG ACC TAG ACG AG	GAT GCA AAG GTC GTT GTC CT	152
COL1A1	CAG GAA TTC GGC TTC GAC	CCA TGT GAA ATT GTC TCC CAT T	164
COL11A1	CAC ATG GCA AAA GCT TTG AA	TGG ATG GAT GAG AAT GAG CA	185
COL15A1	TGA ACC TCA AGG GCC AAG TA	TTC GCC ATG CTT CAC AGT AG	204
COL3A1	AAA GAC GCA TGT TAT GGT GCT	CAG GAT GAA GGA GGA GAA TCC	155
COL1A2	CAC CAC TTG TGG CTT TTG AA	CAA AAC AAG GAC CTC AGT TCA TC	151
HMOX1	ATG ACA CCA AGG ACC AGA GC	GTG TAA GGA CCC ATC GGA GA	153
JUNB	ACC CCT ACC GGA GTC TCA AA	GTT GCT GTT GGG GAC AAT CA	155
THBS4	TAT CGC TGC AAT GAC ACC AT	TTG CCA CAT TCA CAT AAA ACG	194

**Table 3:** List of 16 condensed genes which are differentially expressed in all patient samples, including the pool of RNA obtained from eight AAD patients.

Name of the most upregulated genes	Median expression
Early growth response 1	2.87
Interleukin 6	1.32
Integrin, alpha 5	2.11
Jun B proto-oncogene	1.89
Chemokine (C-C motif) ligand 2	2.49
FBJ murine osteosarcoma viral oncogene homolog B	1.84
Matrix metalloproteinase 19	1.96
**Name of the most downregulated genes**	
Fibulin 1	−2.56
Decorin	−3.47
Fibulin 2	−0.32
Thrombospondin 4	−2.18
Proteoglycan 4	−1.89
Microfibrillar associated protein 5	−0.28
Chemokine (C-X-C motif) ligand 14	−2.74
Secreted frizzled-related protein 2	−2.18
Chemokine (C-C motif) ligand 13	−2.47
Small inducible cytokine b14 precursor	−2.74
Chemokine (C-C motif) ligand 15	−3.06

**Table 4:** A sublist of 32 genes was created from genes, whose median values were significant and above the threshold of two-fold regulation. These genes are clustering in a group assigned to ‘extracellular space.’ Nine genes, identical to those represented in Table 3, are marked in cursive.

Name	Description
C2	complement component 2
PMP22	peripheral myelin protein 22
SDC1	syndecan 1
COL11A1	collagen, type XI, alpha 1
ITGB4	integrin, beta 4
DCN	decorin
*THBS4*	thrombospondin 4
COL1A1	collagen, type I, alpha 1
IFIT1	interferon-induced protein with tetratricopeptide repeats 1
LUM	lumican
MMP9	matrix metalloproteinase 9
COL3A1	collagen, type III, alpha 1
COL1A2	collagen, type I, alpha 2
JAK1	Janus kinase 1
MMP12	matrix metalloproteinase 12
TNFSF10	tumour necrosis factor (ligand) superfamily, member 10
HSPA4	heat shock 70 kDa protein 4
*FBLN2*	fibulin 2
*SFRP2*	secreted frizzled-related protein 2
COL15A1	collagen, type XV, alpha 1
*FBLN1*	fibulin-1
GEM	GTP binding protein
MATN2	matrilin 2
CCL21	chemokine (C-C motif) ligand 21
CCL19	chemokine (C-C motif) ligand 19
SLCO2A1	solute carrier organic anion transporter family, member 2A1
MFAP5	microfibrillar-associated protein 5
*CXCL14*	chemokine (C-X-C motif) ligand 14
*PRG4*	proteoglycan 4
*CCL13*	chemokine (C-C motif) ligand 13
*CCL14*	chemokine (C-C motif) ligand 14
*CCL15*	chemokine (C-C motif) ligand 15
